# Gelatin-Based Hybrid Scaffolds: Promising Wound Dressings

**DOI:** 10.3390/polym13172959

**Published:** 2021-08-31

**Authors:** Sindi P. Ndlovu, Kwanele Ngece, Sibusiso Alven, Blessing A. Aderibigbe

**Affiliations:** Department of Chemistry, University of Fort Hare, Alice 5700, South Africa; 201304407@ufh.ac.za (S.P.N.); 201102901@ufh.ac.za (K.N.); 201214199@ufh.ac.za (S.A.)

**Keywords:** wound care, wound dressings, polymers, gelatin, hydrogels, nanofibers, sponges

## Abstract

Wound care is a major biomedical field that is challenging due to the delayed wound healing process. Some factors are responsible for delayed wound healing such as malnutrition, poor oxygen flow, smoking, diseases (such as diabetes and cancer), microbial infections, etc. The currently used wound dressings suffer from various limitations, including poor antimicrobial activity, etc. Wound dressings that are formulated from biopolymers (e.g., cellulose, chitin, gelatin, chitosan, etc.) demonstrate interesting properties, such as good biocompatibility, non-toxicity, biodegradability, and attractive antimicrobial activity. Although biopolymer-based wound dressings display the aforementioned excellent features, they possess poor mechanical properties. Gelatin, a biopolymer has excellent biocompatibility, hemostatic property, reduced cytotoxicity, low antigenicity, and promotes cellular attachment and growth. However, it suffers from poor mechanical properties and antimicrobial activity. It is crosslinked with other polymers to enhance its mechanical properties. Furthermore, the incorporation of antimicrobial agents into gelatin-based wound dressings enhance their antimicrobial activity in vitro and in vivo. This review is focused on the development of hybrid wound dressings from a combination of gelatin and other polymers with good biological, mechanical, and physicochemical features which are appropriate for ideal wound dressings. Gelatin-based wound dressings are promising scaffolds for the treatment of infected, exuding, and bleeding wounds. This review article reports gelatin-based wound dressings which were developed between 2016 and 2021.

## 1. Introduction

Wound care is a concern globally with various challenges including the increasing prevalence of type II diabetes, obesity, an aging population, and the need for cost-effective wound dressings [1,2]. The wounds are generally classified based on their healing process as acute or chronic wounds. Acute wounds are lesions that heal within the expected timeframe of approximately 2–3 months depending on the depth and size of the injury in the skin [3]. Chronic wounds fail to heal through the ordinary wound healing process over a prolonged period. Examples of chronic wounds include diabetic wounds, ulcer wounds, burn wounds, etc. [4,5]. All types of wounds require good clinical care to prevent delayed wound healing processes that may be caused by microbial infections and other negative factors. More than 300 types of wound dressing products are available in the market. However, one wound dressing is not appropriate for the treatment of all wound types [6]. In the United States of America (USA), a yearly cost of 20 billion dollars is spent on the wound care of chronic injuries [7]. The global market cost of chronic wound care was 10.12 billion dollars in 2019, and it is projected that the cost will increase to 16.36 billion dollars in 2027 [8]. These statistics demonstrate the negative socio-economic impacts of wound care globally, indicating an urgent need to develop affordable wound dressings for effective wound care.

**Figure 1:**
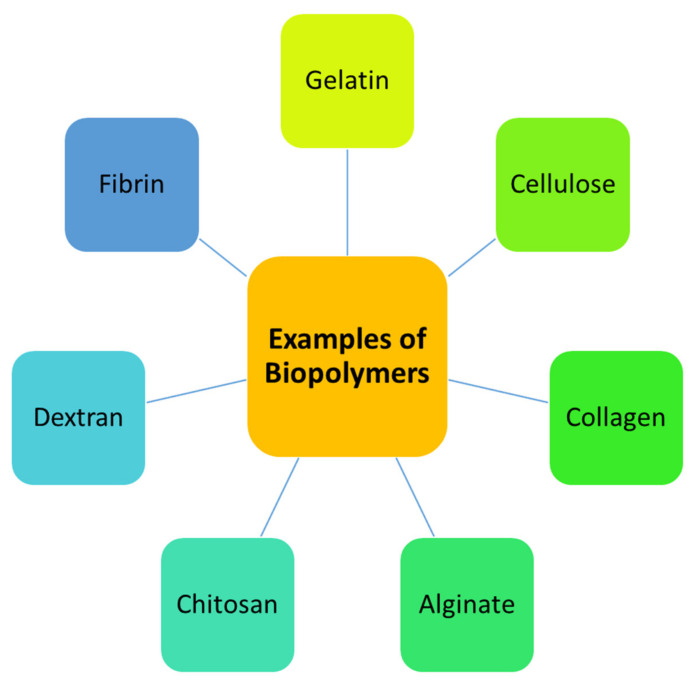
Examples of Biopolymers in Biomedical applications.

The properties of an ideal wound dressing that make it suitable to provide a proper environment for the healing process include durability, flexibility, permeability to water vapor, adherence to the tissue, and good mechanical properties [9]. Furthermore, the dressing materials should hydrate/dehydrate the wound, maintain a moist environment, protect the wound from infections, and prevent maceration [10]. Polymer-based wound dressing materials can provide the aforementioned properties. Polymers that can be used for the fabrication of dressings are mainly classified as biopolymers and synthetic polymers [11]. Examples of biopolymers include gelatin, cellulose, chitin, alginate, hyaluronic acid, chitosan, dextran, elastin, fibrin, etc. (Figure 1) [12]. The wound dressings that are formulated from these polymers usually suffer from poor mechanical properties. The combination of biopolymers with synthetic polymers is a promising design strategy to overcome the poor mechanical properties of biopolymer-based wound dressings.

Gelatin is a biopolymer with interesting properties that have greatly attracted the attention of many biomedical researchers, such as low antigenicity, good biodegradability, and biocompatibility in the physiological environment [13]. The gelatin-based materials offer excellent characteristics of wound dressings. The fast degradation time and highly hydrophilic surface make gelatin inappropriate as a base material for the development of wound dressings. Thus, gelatin is combined with other polymers, especially synthetic polymers [14]. This review will discuss the outcomes of gelatin-based hybrid dressings for wound care.

## 2. Phases of Wound Healing Process

**Figure 2:**
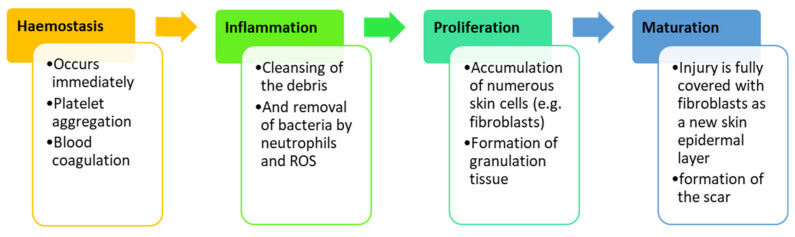
Sequential Phases of Wound healing.

Wound healing is an important physiological and complex process, involving a multifaceted process, including the hemostasis phase, inflammation phase, proliferation phase, and maturation phase (Figure 2) [5]. The hemostasis phase occurs immediately after the injury and the exposed collagen, sub-endothelium, and tissue factor trigger platelet aggregation that results in degranulation and the release of growth factors (GFs) and chemokines to form the blood clot [15]. Hemostasis usually occurs concurrently with the inflammation phase. In the inflammation phase, neutrophils at the wound site cleanse the debris and remove bacteria together with reactive oxygen species (ROS), thereby offering an appropriate environment for the wound healing process [16]. The injury site tends to become red, swollen, and warm due to the presence of white blood cells [16].

The third phase of wound healing is the proliferation phase, and this phase is characterized by an accumulation of numerous cells and profuse connective tissue. The injury encompasses endothelial cells, keratinocytes, and fibroblasts. Extracellular matrix (ECM) components, including collagen, hyaluronic acid, proteoglycans, and elastin produce a granulation tissue to substitute the original development of blood clots [17]. Many varieties of cytokines and GFs that are involved in this phase include transforming growth factor-β family (TGF-β, including TGF-β1, TGF-β2, and TGF-β3), interleukin (IL) family, and angiogenesis factors (e.g., vascular epidermal growth factors (VEGFs)), result in the formation of new tissue [18]. The new tissue is normally pink or red when it covers the wound site. The last phase of the wound healing process is the maturation phase, which requires a precise balance between the apoptosis of the remaining cells and the formation of new cells [18]. Gradual degradation of profuse ECM and the immature type III collagen and development of mature type I collagen is critical in this phase. The surface of the injury is fully covered with fibroblasts as a new epidermal layer of the skin, and the formation of the scar occurs [19]. The maturation phase usually occurs within few months or even years. Any disruption in the wound healing phases can result in the formation of keloids or chronic wounds [18].

## 3. Classification of Wound Dressings

**Figure 3:**
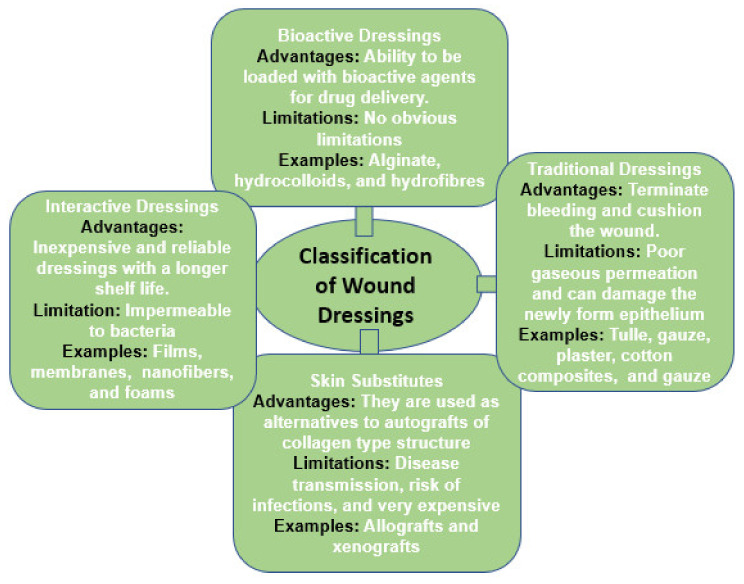
Classification of Wound Dressings.

Wound dressings are materials used to protect a wound. They also act as a barrier against pathogens. An ideal wound dressing offers good features, such as good applicability, biocompatibility, stability, and flexibility, as well as ensuring a good gas barrier and biodegradability to speed up the healing process and reduce the risk of infections. Wound dressings must also be able to manage wound exudates to prevent bacterial invasion [20]. Wound dressings are categorized as primary and secondary dressings. The primary wound dressings are applied directly to the wounded area, while the secondary dressing is used to cover the primary wound dressing [21]. There are various applications of wound dressing materials. Hence, they are classified into four groups, namely traditional/passive dressings, skin substitutes, interactive/artificial dressings, and bioactive dressings (Figure 3).

Traditional/passive wound dressings are usually used in the first step of treatment to stop bleeding and prevent further interaction of the wound with the environment [22]. These dressings have disadvantages, e.g., they can cause bleeding, display poor permeation of vapor, and damage the newly formed epithelium after removal. Bacterial infections can also result from the leakage of exudates from these dressings. The traditional dressing examples are tulle, gauze, and gauze cotton composites, which are distinguished by high absorption capacity [23].

Skin substitutes are biological dressings and they are additionally classified as allografts, xenografts, and tissue derivatives. Allografts are fresh or freeze-dried skin fragments collected from donors and their use is limited by immune reactions, resulting in rejection by the body. Disadvantages of allografts include disease transmission, risk of infections and they are very expensive with limited shelf life. A xenograft is a tissue graft or organ transplant from the recipient’s donor of a different species (for example from animals to humans) [7].

Interactive/artificial wound dressings are frequently formulated from synthetic polymers and biopolymers. The most widely used biopolymers are gelatin, alginate, chitosan, etc. Artificial wound dressings can be classified as foams, films, composites, sprays, etc. They consist of mostly transparent polymeric films and shapes, permeable to water vapor and oxygen, but impermeable to bacteria. These dressings are suitable for less exuding wounds [23]. The advantages of interactive wound dressings are that they are inexpensive and reliable dressings with a longer shelf life [22]. Bioactive wound dressings are prepared from biopolymers and are encapsulated with bioactive agents, such as antimicrobials and growth factors, to enhance the wound healing process. Examples of biopolymers are collagens, alginate, hydrocolloids, and hydrofibres [23].

## 4. Properties of Gelatin in Wound Dressing Applications

**Figure 4:**
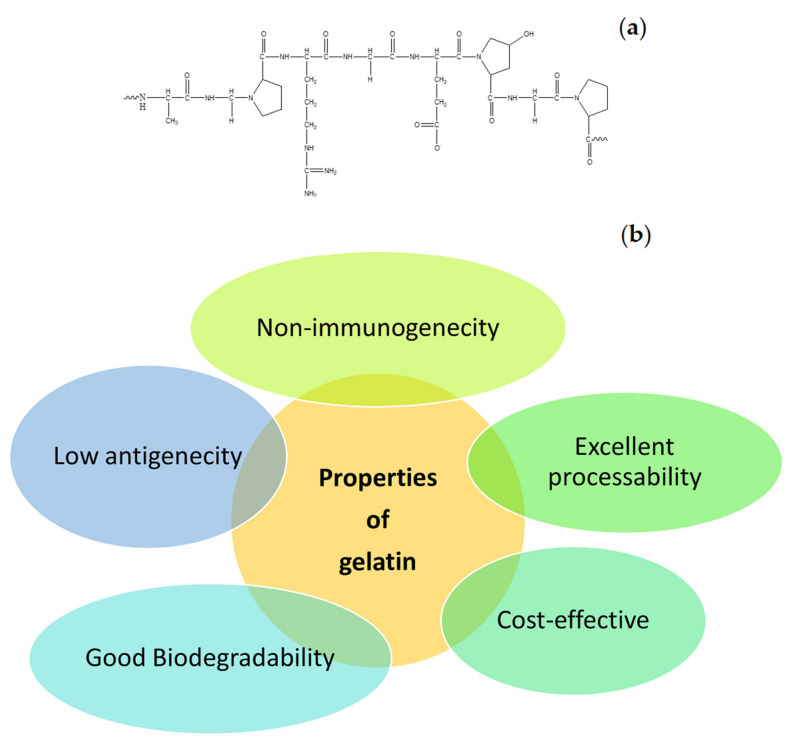
(**a**) Basic molecular structure of gelatin (**b**) Properties of gelatin.

Many natural polymers are frequently used in the formulation of wound dressings. These polymers include gelatin, cellulose, alginate, collagen, elastin, chitosan, chitin, dextran, etc. The common interesting properties of natural polymers are good biocompatibility and biodegradation, non-toxicity, non-immunogenicity, and affordability. In addition, some of the natural polymers exhibit strong attachment to injured tissues and stimulate blood coagulation, accelerate the wound healing process, and induce skin regeneration [12]. Gelatin is one of the biopolymers that is commonly utilized in the design of wound dressings. It is also utilized for biomedical and pharmaceutical applications [24,25]. The molecular structure of gelatin is shown in Figure 4a. It is a natural mimic of the extracellular matrix (ECM) of human tissues and organs. It is broadly utilized in the field of tissue engineering [26]. The properties of gelatin that have been attracting the attention of most biomedical researchers include excellent biocompatibility, good biodegradability, cell-interactivity, non-immunogenicity, as well as its excellent processability, ready availability, and cost-effectiveness (Figure 4b) [27]. The pretty low antigenicity of gelatin also makes it a well-established biopolymer used in numerous biological applications. However, gelatin is a hydrophilic protein, and crosslinking is normally required to enhance its mechanical performance and stability, making gelatin materials insoluble in biological environments [28]. Numerous gelatin crosslinking procedures are available, such as enzymatic using transglutaminase, or chemical using fructose, diepoxy, genipin, dextran dialdehyde, formaldehyde, diisocyanates, glutaraldehyde, or carbodiimides [13].

In various studies, gelatin biopolymers were designed as films, gels, powders, or scaffolds for haemorrhage control in numerous surgical methods [29]. Porous gelatin matrices absorb wound exudates and maintain moisture, thus promoting the wound healing process. Gelatin-based dressings act as porous materials for cell migration and offer mechanical and structural support for the development of new tissue [30]. Although gelatin is a promising biopolymer employed as a wound dressing material, it has no antibacterial efficacy to prevent wound infections or bacterial invasion of the wound [31]. It is combined with other polymers to produce hybrid polymers with superior antibacterial effects.

Gelatin-based scaffolds can be loaded with various antimicrobial agents, such as metal-based nanoparticles, antibiotics, phytochemicals (e.g., curcumin), plant extracts (e.g., Aloe vera), etc., to overcome their poor bactericidal effects [31]. To the best of our knowledge, only two gelatin-based wound dressing materials are commercially available: Gelfoam and Surgifoam. Gelfoam and Surgifoam are composed of porcine gelatin and collagen. Gelfoam and Surgifoam are in the form of compressed sponge and sponge, respectively [32,33]. These commercially available gelatin dressings demonstrate outstanding hemostatic effects. Hence, they are very suitable for bleeding wounds. However, they suffer from some shortcomings, including non-elasticity, etc. [33].

## 5. Gelatin-Based Hybrid Wound Dressings

### 5.1. Hydrogels

**Figure 5:**
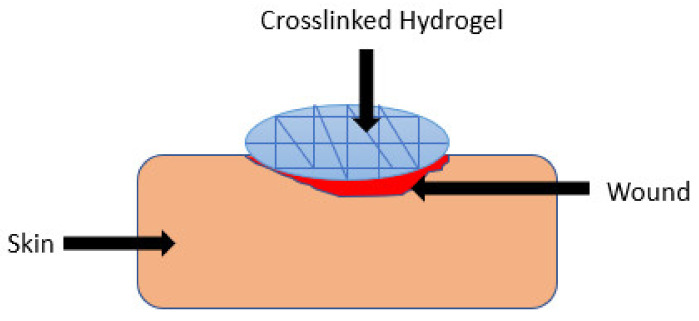
Crosslinked Hydrogel on Skin Wound.

Hydrogels are polymeric materials with a good hydrophilic composition that enables their high retention of a significant quantity of water and other biological fluids within their three-dimensional network (Figure 5) [34]. They can be modified for enhanced stability or degradation in the event of contact with biological fluids over an extended period. These polymeric materials have been used in wound healing applications due to their biodegradation, biocompatibility, porosity, ability to encapsulate and release bioactive agents, flexibility, and high-water content [35]. The other advantages of polymer-based hydrogels that have attracted a lot of attention among biomedical researchers in the field of wound management include patient compliance, accelerated wound healing mechanism, the high adsorption capacity of biological fluids which provide moisture to the wound bed, their capability to protect the wound from microorganisms, and specific environmental stimuli-responsiveness (e.g., pH, temperature, and ionic strength). The environmental stimuli-responsive nature of the wound dressings promotes drug release into the infected wound area in a sustained profile, thereby reducing the dosing frequency [36,37]. Several researchers have reported gelatin-based hybrid hydrogels (Table 1).

Hsu et al., formulated gelatin-hyaluronic acid (HA) hybrid hydrogels encapsulated with recombinant thrombomodulin by chemical cross-linking followed by freeze-drying for diabetic wound management [38]. The scanning electron microscopy (SEM) images demonstrated porous morphology, indicating the porosity of the hydrogels was decreased by an increase in the HA content. The water absorption of the hybrid hydrogels rapidly increased within the first 30 min, and the hydrogels swelled more than 11-fold within 24 h, which is beneficial for drug absorption, absorbing wound exudates, and offering a moist environment for injury bed. The wound closure studies in vivo using streptozotocin-induced mice showed that the thrombomodulin-loaded hybrid hydrogels significantly accelerated wound contraction after two days of wounding when compared to pristine hybrid hydrogels [38]. Mao et al., fabricated gelatin-oxidized starch hybrid hydrogels for wound healing applications. The in vitro cytotoxicity studies demonstrated the high cell viability of skin fibroblasts (L929 cells) of the hydrogels, indicating the good biocompatibility and non-toxicity of the hydrogels. The in vivo wound healing studies demonstrated a fast wound healing process with less scar formation when the wounds in the rabbit model were treated with hybrid hydrogels [39].

Dang et al., developed injectable hybrid hydrogels that are based on gelatin and pluronic. They were loaded with nanocurcumin for burn wound care. The in vitro cytocompatibility analysis demonstrated that all the hydrogel formulations presented no toxic effect on the fibroblasts after 48 h of incubation, suggesting good cytocompatibility of the hybrid hydrogels. The burn wounds on mice models were almost closed on day 10 when treated with nanocurcumin-loaded gelatin-pluronic hydrogels compared to the pristine hydrogels and other commercial dressings [40]. Zheng et al., prepared injectable hydrogels using gelatin and gellan loaded with tannic acid for antibacterial wound dressing. The in vitro antimicrobial analysis of tannic acid-loaded hybrid hydrogels using agar disc diffusion showed superior antibacterial efficacy against *S. aureus*, *E. coli*, and drug-resistant bacteria (methicillin-resistant *S. aureus* [MRSA]). The in vivo studies on full-thickness wounds on mice model showed that the wounds treated with tannic acid-loaded hydrogels were significantly healed faster and they were fully closed on the 12th-day post-surgery without scar development [41]. Dong et al., designed injectable PEG-gelatin hydrogels encapsulated with adipose-derived stem cells (ASCs) for cutaneous wound management. The cytotoxicity analysis revealed more than 85% cell viability of ASCs when encapsulated in hybrid hydrogels, suggesting that the hydrogels can be useful for the delivery of numerous cells for in vivo and clinical application. The in vivo studies using murine excisional wound healing model demonstrated that the wounds treated with ASC-loaded hydrogels were completely closed on day 10 while the wounds treated with plain ASCs and plain hydrogels were closed on days 12 and 16, suggesting that the encapsulation of ASCs in the hydrogels significantly accelerated the wound healing process [42].

Khamrai et al., prepared a gelatin-based polyelectrolyte hydrogel patch loaded with curcumin for wound healing using a sequential solution mixing and casting method. The prepared hydrogel displayed self-healing capability at a physiological pH of 7.4 and acted as a transdermal drug delivery system of curcumin [43]. The gelatin-based hydrogel patch was loaded with ionically self-assembled bacterial cellulose obtained from *Glucanoacetobacter xylinus* bacterial strain. The mechanical properties exhibited by the gelatin-based hybrid hydrogel showed an increased elongation of 4.8% and a decreased modulus of 4.6 MPa, stating that the loaded bioactive agent had a positive effect on the elongation and a negative effect on the modulus. Atomic force microscopy (AFM) depth profilometry analysis showed complete healing after application in a buffer of pH 7.4 after damage [43]. The in vitro drug release profile displayed a controlled release of curcumin from the prepared gelatin-based hydrogel, which contributed to the antimicrobial activity of the hydrogel, enhancing wound healing. The in vitro antimicrobial studies demonstrated that curcumin-loaded hydrogels possessed enhanced antibacterial activity against *E. coli* and *S. aureus* when compared to plain hydrogels, indicating their potential use for the treatment of bacterial-infected wounds [43].

Treesuppharat et al., formulated gelatin-bacterial cellulose hydrogels encapsulated with methylene blue via a copolymerization process and were cross-linked using glutaraldehyde [44]. The hydrogel composites were malleable and flexible. The SEM images of hybrid hydrogels revealed a porous morphology, suggesting good porosity for effective drug delivery. The results from AFM agreed with the SEM morphology. The prepared hybrid hydrogel showed good thermal stability, mechanical properties, and chemical resistance before the loading of bacterial cellulose. After loading the hydrogels with bacterial cellulose, their porosity changed from macro-porous to mesoporous pores. The swelling studies demonstrated the hydrogel network swelling capacity in the range of 400–600% [44]. 

Li et al., prepared gelatin-based composite hydrogel by gels spinning with PEG6000 as a modifier and cross-linked with dialdehyde carboxymethyl cellulose (DCMC) [45]. The prepared samples of the gelatin-based hydrogels were named as follows GeP, GeP-D1000, GeP-D500, GeP-D100, and GeP-D50 when the ratio of DCMC to gelatin was 0, 1/1000, 1/500, 1/100, and 1/50, respectively. The mechanical properties for the gelatin-based hydrogel fibres showed the highest tensile strength of 2.15 + 0.21 MPa and a reduced elongation at a break of 10.2 + 0.8% for GeP-D50. The increase in the tensile strength was attributed to an increase in DCMC in the hydrogel, resulting in a decrease in the elongation at break. The gelatin-PEG hydrogel fiber showed excellent swelling behavior in the range of 89–93%, stating that it can absorb wound exudates, and provide a moist environment to accelerate the wound healing process. The DCMC cross-linker reduced the swelling degree of hydrogels, which is advantageous to the hydrogel fibres to prevent the undesired reduction of the mechanical properties. The SEM of the prepared hydrogel fibres displayed a porous network and 3-D morphology which indicates that the hydrogel will aid in cell adhesion and proliferation. The hydrogel with a more porous network was observed to be GeP-D1000, meaning it can hold free water compared to GeP-D50 because it is more compact. The hydrogel fibers GeP-D500, and GeP-D1000 were biocompatible when evaluated in normal fibroblasts with no visible dead cells. However, GeP-D50 and GeP-D100 were not biocompatible [45].

Fan et al., fabricated gelatin-PVA-chitosan hybrid hydrogels using the gamma irradiation method for wound dressing application [46]. The swelling analysis revealed that gelatin-PVA-chitosan hydrogels showed excellent swelling behavior, which was attributed to an increase in the ratio of gelatin and chitosan. Their good swelling property was attributed to the hydrophilic nature of gelatin and PVA polymers. The hybrid hydrogels demonstrated good capability to maintain a moist environment by retaining 10-20% of water, meaning it has a good water evaporation rate. The mechanical properties for the gelatin-PVA-chitosan hydrogels revealed that the highest tensile strength was 2.2 MPa for the hydrogel with a ratio weight of chitosan: gelatin (1:3). The elongation of the prepared hydrogel decreased with an increase in gelatin, while the tensile strength increased with an increase in gelatin, which enhanced the mechanical properties of the hybrid hydrogels. The SEM images showed a porous morphology for the gelatin-PVA-chitosan hydrogels, revealing that the hydrogels are permeable to wound exudates and water vapor. The gelatin-PVA-chitosan hydrogel displayed a good blood clotting with the lowest BCI index of 0.032 for the hydrogel with a ratio weight of chitosan: gelatin (1:1) [46].

The gelatin-based hybrid hydrogels display interesting features, as reported in a series of in vitro and in vivo studies by some researchers. Their interesting features make them promising scaffolds for wound dressings. The blending of gelatin with other polymers, especially synthetic polymers, for the fabrication of hydrogels resulted in an excellent mechanical performance which is beneficial for easy handling and application during wound care. The mechanical properties of gelatin-based hybrid hydrogels imitate those of human skin, suggesting their compatibility with the skin. SEM micrographs of hybrid hydrogel scaffolds showed a porous morphology that can improve hydrogel swelling capacity, absorption of wound exudates, and proliferation and adhesion of skin cells that are essential during the wound healing process. The loading of bioactive agents (e.g., antibiotics and antioxidant agents) into the gelatin hybrid hydrogels significantly improved their biological activities, resulting in accelerated wound healing in vivo. These hydrogels displayed controlled drug release that can further enhance their biological activities and reduce drug toxicity.

### 5.2. Films and Membranes

**Figure 6:**
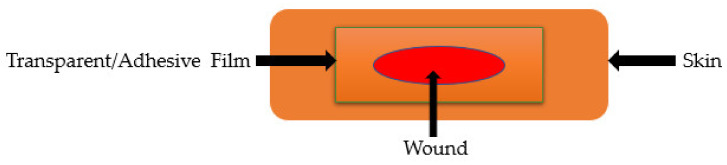
Transparent Film Dressing on Skin Wound.

Films are semi-permeable dressings with a translucent and adhesive nature (Figure 6). Films provide a moist environment, ease cell migration, promote autolysis, are partially permeable to water vapor and oxygen, and inhibit bacteria proliferation [47]. They are suitable for treating chronic wounds (on the heels, elbow, and a flat surface of the body), moderate exuding wounds, and superficial wounds. Films are cost-effective, clear necrotic debris, are waterproof, and permit cyclic inspections of a wound [48]. The disadvantage of film dressings is that they cause skin maceration upon removal and most of them are non-absorptive or less absorptive. The film dressings are not frequently changed, depending on the amount of exudates, they can be changed once a week [46]. Gelatin-based hybrid film wound dressings have been reported by several researchers (Table 2). Taheri et al., formulated gelatin-chitosan hybrid films encapsulated with tannic acid and/or bacterial nanocellulose for wound healing applications [49]. The FTIR and XRD analysis confirmed the successful preparation of hybrid films by displaying the expected peaks. The SEM images exhibited scattered white spots, indicating the desired dispersion of nanocellulose particles on the surface of the films. The water vapor transmission experiments demonstrated that the addition of tannic acid and nanoparticles in the films significantly decreased the WVTR, providing sufficient moisture for wound healing. The results from the mechanical analysis showed that all the films possessed a tensile strength of more than 80 MPa. The in vivo studies using Wistar rats demonstrated that the full-thickness skin wounds treated with the dual drug-loaded films and nanoparticle-loaded films were healed with a closure faster than the tannic acid-loaded films and plain hybrid films [49]. Sakthiguru and Sithique designed gelatin-chitosan biocomposite films incorporated with allantoin for wound healing application [50]. The water absorption tests showed enhanced water absorption capacity of allantoin-incorporated hybrid films. The in vitro cytotoxicity experiments of all the films assessed using the MTT assay demonstrated more than 80% cell viability when incubated with L929 fibroblasts, revealing excellent biocompatibility and non-toxicity. The antimicrobial studies demonstrated that allantoin-loaded films had superior antibacterial activity against *S. aureus* and *E. coli* when compared to the free allantoin and plain films, indicating that the allantoin-incorporated dressings are potential antibacterial wound dressing materials [50].

Akhavan-Kharazian and Izadi-Vasafi developed hybrid films that are prepared from gelatin and chitosan. The films were incorporated with nanocrystalline cellulose and calcium peroxide for wound healing [51]. The swelling analysis demonstrated high swelling behavior at pH 5, 7, and 9, simulating wound exudate, physiological condition, and an infected wound, respectively. The water vapor permeation studies of the hybrid films prepared exhibited WVTR values in the range of 35 to 45 g/m^2^/h which is appropriate for maintaining suitable fluid balance on the wound bed. The in vitro cytotoxicity analysis using the MTT assay showed almost 100% cell viability of the human fibroblast cells when immersed with dual-loaded film/ plain films for seven days, showing that the prepared gelatin-based hybrid films are non-toxic [51]. Patel et al., prepared gelatin-chitosan films for drug delivery of lupeol for wound dressing application. The SEM micrograph images of the hybrid films showed relatively smooth, fibrous, and porous morphology appropriate for increasing the oxygen supply to the injury for accelerated wound healing. The in vitro drug release studies showed that the release of lupeol from the films followed a biphasic release pattern with an initial burst release followed by a sustained release of 90.99 ± 1.27% lupeol after 24 h. The in vitro antioxidant experiments demonstrated that the incorporation of lupeol in the films significantly enhanced the free radical-scavenging effects of the gelatin-based hybrid films, revealing the wound healing properties of lupeol-loaded films at the inflammation phase of the wound healing [52].

Türe reported gelatin-alginate composite films loaded with hydroxyapatite for wound management. The in vitro antimicrobial studies of the drug-loaded films using agar disc diffusion assay demonstrated that the drug-loaded films were effective in inhibiting the growth of *S. aureus* and *E. coli* while the films not loaded with the drug did not exhibit any inhibitory effect, suggesting that the drug-loaded hybrids are potential dressings for the treatment of bacteria-infected wounds [53]. Cahú et al., developed gelatin-chitosan-chondroitin 4-Sulfate hybrid films incorporated with zinc oxide (ZnO) nanoparticles. The nanoparticles-loaded films did not show any toxicity towards the skin fibroblasts (3T3) or keratinocytes (HaCaT) cell lines, indicating excellent biocompatibility with good antibacterial effect against *S. aureus* and *E. coli* in vitro. The in vivo wound healing experiments using the rat model demonstrated that gelatin-based hybrid films significantly increased the percentage of wound reduction from 65% to 86% in full-thickness excision compared with only 51% for the control, after six days [54]. Evranos et al., prepared bone ash-incorporated gelatin-chitosan film wound dressings loaded with ciprofloxacin. The in vitro drug release studies at physiological conditions showed a controlled release of ciprofloxacin from the films due to the incorporation of bone ash into the hybrid films. These films demonstrated superior antimicrobial effects against *E. coli* and *Bacillus subtilis* (*B. subtilis*) [55].

Baek et al., developed gelatin-PCL-(+)-catechin films for wound care. The FTIR data confirmed the successful formulation of films, while the in vitro cytotoxicity analysis demonstrated good cell viability and proliferation of NIH/3T3 cells, indicating good biocompatibility that is appropriate for an ideal wound dressing [56]. Garcia-Orue reported gelatin-chitosan bilayer hydrofilms for wound healing application. The water uptake studies of the hybrid hydrofilms displayed a significantly higher swelling ability of approximately 700% in equilibrium. The water vapor transmission of the films demonstrated a WVTR value of 787.0 ± 50.9 g/m^2^·day. The cytotoxicity studies demonstrated more than 70% cell viability of fibroblast cells when immersed with the hydrofilms, revealing good biocompatibility. The ex vivo experiments using human skin demonstrated accelerated wound contraction of the wounds treated with the hydrofilms [57].

Bhowmik et al., prepared gelatin-based biocomposite films incorporated with crystalline cellulose (CCs) for wound healing application [58]. The SEM images of the gelatin-CCs films displayed a rough morphology with a lot of crystals on the surface of the films. The mechanical characterization of the gelatin-CCs films in the ratio of 10:10 showed the highest tensile strength of 64.16 MPa with a tensile modulus of 2.64 MPa. The tensile strength and tensile modulus increased with an increase in the concentration of CCs. The gelatin-CCs films demonstrated an excellent fluid absorbing capability, making the films suitable for maintaining a moist environment on the wound area, which is essential for wound healing. The gelatin-CCs films were biocompatible and non-toxic. The in vivo wound healing studies and histological analysis studies using the mice model demonstrated that, on the 10th day, the wound dressed with gelatin-CCs films contracted with re-epithelization on the wound bed without inducing trauma on the wound bed [58].

On the other hand, membranes display similar properties as film wound dressings. The advantages of polymer-based membranes in wound dressing include their ability to absorb excess exudates, maintain appropriate moisture for the wound healing process, retain biological fluids under pressure, do not require frequent dressing changes, reduces the disruption of the wound bed, and present potential cleaning activity [59]. Furthermore, membranes demonstrate good mechanical properties, such as softness, comfortability, flexibility, and stretchability [59].

Xu et al., designed gelatin-CM and chitosan-HA membranes for corneal wound healing applications. The cytocompatibility analysis of the membranes revealed high cell viability and proliferation of the primary rabbit corneal epithelial cells, revealing good cytocompatibility. The in vivo studies using alkali-induced corneal damage in rabbits demonstrated that these hybrid membranes could significantly enhance corneal epithelial reconstruction and restore cornea transparency and thickness [60]. Kenawy et al., prepared biodegradable cinnamaldehyde-crosslinked gelatin-chitosan membranes for wound healing applications. The wettability studies demonstrated a water contact angle of about 29°, indicating the hydrophilic nature of the membranes. The in vitro antimicrobial experiments demonstrated a higher growth inhibition against *Pseudomonas aeruginosa (P. aeruginosa*), *Salmonella*, *E. coli*, and *S. aureus* for cinnamaldehyde-crosslinked gelatin-chitosan membranes than the uncrosslinked hybrid membranes [61].

Shi et al., prepared a gelatin elastomer nanocomposite membrane incorporated with polymyxin B sulfate and ciprofloxacin, loaded with halloysite clay nanotubes (HNTs) using a simple gelling method and a hot melt blending technique [62]. The mechanical analysis showed a tensile strength of 1 MPa, while the increase in the amounts of HNTs led to an increased tensile strength of 2 MPa. The prepared elastomer nanocomposite membranes were capable of protecting and promoting wound healing. The elastomer nanocomposite membranes displayed a good water uptake of over eight times its swelling weight. The HNTs enhanced the properties of the prepared membrane and affected the water absorption of the samples. The in vitro cytotoxicity studies of the nanocomposite membranes displayed biocompatibility and non-toxicity on L929 cells. The elastomer nanocomposite membrane displayed a sustained release of HNTs. The bacterial zone of inhibition of all the elastomer membranes was significant against *P. aeruginosa* and *S. aureus* [62].

Gelatin-based hybrid films and membranes exhibit good mechanical properties that make them easy for application on wounds. Most of the films demonstrated moderate WVTR appropriate to prevent wound dehydration and provided a suitable moist environment for the wound healing process. The high cell viability and proliferation of various skin cells, when incubated with gelatin films or membranes, confirmed the excellent biocompatibility and non-toxicity of the gelatin-based hybrid materials. The combination of gelatin with other polymers such as chitosan for the fabrication of films and membranes resulted in high growth inhibition of various bacterial strains that are responsible for bacteria-infected wounds. Generally, films display low absorption capacity, making them inappropriate for exuding wounds. However, gelatin-based hybrid films exhibited higher absorption capability due to the hydrophilic nature of gelatin. The drug-loaded gelatin films and membranes significantly resulted in good biological effects, such as antibacterial and antioxidant activities, that are required in wound treatment, especially for chronic wounds.

### 5.3. Sponges

**Figure 7:**
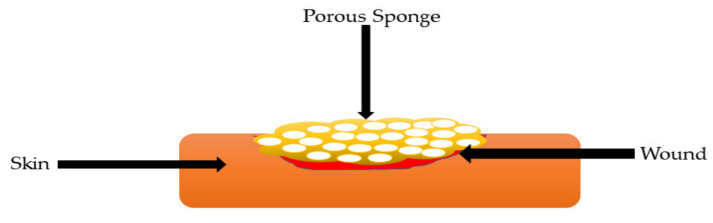
Microporous Sponge on Skin Wound.

Sponges are soft and flexible materials with a well interconnected micro-pore structure (Figure 7). Due to their unique structural features, they have good fluid absorption capability, cell interaction, and hydrophilicity [63]. Their high swelling capacity and quick hemostatic capability make them suitable to prevent the accumulation of exudates. The sponges that absorb a sufficient amount of wound exudates provide a moist environment and also protect the wound bed from bacterial invasion [64]. The porous sponges based on natural as well as synthetic biopolymer have been widely used for biomedical dressings. Sponges with pore sizes between 10 and 100 microns in diameter and interconnected channels increased tissue growth [65]. Gelatin is one of the biopolymers widely reported for the preparation of sponges (Table 3).

Zou et al., reported gelatin-konjac glucomannan hybrid sponges loaded with gold (Au) nanoparticles and gentamicin sulfate for the treatment of bacteria-infected wounds [66]. The transmission electron microscope (TEM) images displayed the shape of Au nanoparticles which was spherical or elliptical in a single dispersed modality with an average particle size of 3.55 ± 0.26 nm. The in vitro cytotoxicity analysis of the hybrid sponges demonstrated a cell viability value of more than 88% on L929 cells, indicating that these dual drug-loaded sponges were non-toxic. The in vitro antimicrobial experiments demonstrated that the dual drug-loaded sponges possessed higher antibacterial efficacy against *E. coli*, *S. aureus*, and MRSA while the plain hybrid sponges did not possess any antibacterial effect against these bacterial strains. The in vivo studies using the rabbit model showed that the full thickness wounds dressed with dual drug-loaded hybrid sponges were almost completely healed on day 14 when compared to other groups [66].

Jinno et al., performed comparison studies of bFGF-loaded gelatin-collagen sponges and collagen sponges in wound healing application. The in vivo wound healing studies using full-thickness skin lesions in rats demonstrated that there were enhancement observed in the dermis-like tissue area, the neoepithelial length, and the angiogenesis rates in the group of animal models treated with bFGF-incorporated sponges compared to collagen sponges and plain gelatin-based hybrid sponges [67].

Ye et al., fabricated tannic acid-crosslinked gelatin-gelatin sponges loaded with Ag nanoparticles for wound healing application. The porosity analysis displayed that the addition of Ag nanoparticles significantly increased the porosity of the sponges from 43.25 ± 3% to 85.13 ± 2%, and this can stimulate the absorption of exudates and allow the exchange of substances between the skin cells. The antimicrobial studies showed that the addition of Ag nanoparticles enhanced the antibacterial effects of the sponges against *S. aureus* and *E. coli*. The in vivo studies using the rabbit model demonstrated that the *S. aureus*-infected full-thickness wounds possessed wound closure rates of 100%, 97%, 90%, and 80% on the 15th day for Ag nanoparticle-loaded sponges, Aquacel^®^ Ag, pristine gelatin-chitosan sponges, and control, respectively [68]. Naghshineh et al., formulated hybrid sponges that are based on gelatin and chitosan. The wound dressings were loaded with curcumin for the treatment of wounds. The in vitro drug release profile showed an initial burst release of curcumin from the sponges followed by a sustained and slow release for 48 h. The in vivo wound closure studies using the rat model showed that the lesions covered with curcumin-loaded sponges were completely healed on day 10 without the formation of scars [69].

Lu et al., examined the effects of chitosan-gelatin sponges loaded with tannins and platelet-rich plasma (PRP) on the rate of wound healing [70]. The FTIR spectra showed that the chitosan and gelatin were crosslinked via chemical bonds, providing the hybrid sponges with good thermostability and appropriate mechanical properties. The porous structure of the sponges conferred the material with good water-absorbing quality. The hybrid sponges inhibited the growth of *E. coli* and *S. aureus* with low toxicity. The wounds covered with the tannins-PRP-loaded sponges healed quicker than the wound covered with pristine gelatin or chitosan sponge in vivo. The addition of PRP to the hybrid sponges accelerated wound healing significantly [70]. Singaravelu et al., formulated gelatin-keratin–fibrin 3D sponges loaded with mupirocin for wound healing. The SEM images of the sponges showed highly porous morphology with randomly interconnected pores that can promote gaseous exchange and cell growth. The in vitro drug release studies demonstrated initial rapid drug release of mupirocin from the sponges followed by a slow and sustained release. The in vitro antimicrobial studies of the drug-loaded sponges against *E. coli* and *S. aureus* displayed a clear zone of inhibition with 8 and 6 mm, respectively, while sponges without mupirocin did not show any inhibition zone [71].

Ye et al., fabricated novel honeycomb-like gelatin-bacterial cellulose composite sponges. The SEM cross-sectional morphology of the fabricated hybrid sponges was a combination of a continuous micrometer-size honeycomb structure with fairly regular, aligned, and straight channels. These hybrid composite sponges had a large surface area and uniform pore distribution, and high porosity with excellent swelling capability. The in vitro drug release studies displayed a sustained release of ampicillin for 48 h that follows a non-Fickian diffusion. The obtained gelatin-based hybrid sponges exhibited excellent antibacterial activity, and good biocompatibility, making them useful in various antibacterial applications [72]. Wen et al., incorporated gelatin into sodium alginate to enhance the shape-forming properties and tetracycline hydrochloride (TCH) was loaded to the fabricated antibacterial gelatin-sodium alginate composite sponges. The swelling analysis of the hybrid sponges displayed good swelling behavior due to their high porosity. The in vitro studies showed that the TCH-loaded sponges exhibited a controlled release profile with good antibacterial efficacy against *E. coli* ATCC25, ATCC922, as well as *B. subtilis* ATCC 9372 and *S. aureus* ATCC 6538. The fabricated gelatin-sodium alginate sponges are potential wound dressings for treating bacteria-infected wounds [73].

Gelatin-based hybrid sponges demonstrate excellent swelling behavior due to their high porosity. The sponges with good swelling behavior are suitable for high exuding wounds. Most of the in vitro drug release profiles of the drug-loaded gelatin-based sponges showed initial burst release followed by a sustained and a slow-release. This mechanism is useful in killing the bacterial strains in the wound bed with continuous protection of the injury from further microbial invasion. Furthermore, the gelatin sponges loaded with bioactive agents exhibited a superior wound healing process without the formation of scars in vivo when compared with some of the commercially available products and pristine gelatin sponges, revealing the efficacy of gelatin-based sponges as potential wound dressings.

### 5.4. Nanofibers and Nanofibrous Materials

**Figure 8:**
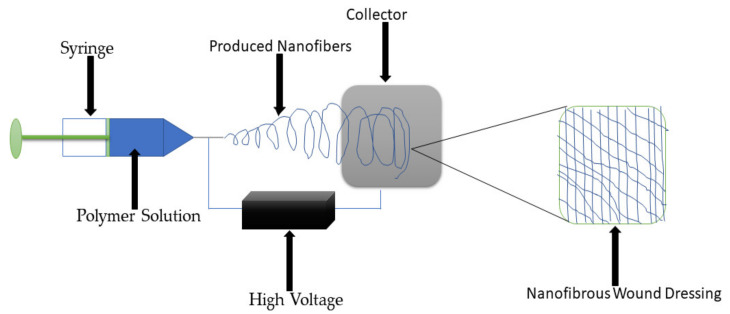
Preparation of Nanofibrous Using Electrospinning Technique.

Nanofibers are wound dressing materials that possess diameter sizes that range from nanometers to a few microns [74]. The popularly used technique for the preparation of these materials is electrospinning (Figure 8) [75]. The electrospun nanofibers are considered appropriate wound dressings for chronic wounds because of their drug delivery capability. Nanofibers imitate the ECM, thereby promoting the proliferation of epithelial cells and the development of new tissues in the wound environment [76]. The advantages of the nanofibers and nanofibrous dressings include their ability to stimulate hemostasis of damaged tissues, promote dermal drug delivery, improve fluid absorption, high-gas permeation, cell respiration, high surface area to volume, high porosity, maintaining of the moist environment, thereby inhibiting microbial infections [77,78]. Many gelatin-based hybrid nanofibers have been reported by researchers for the treatment of different wounds (Table 4). Ajmal et al., formulated gelatin-PCL hybrid nanofibers co-loaded with quercetin and ciprofloxacin hydrochloride for wound healing application. The SEM micrographs of dual drug-loaded nanofibers displayed uniform size distribution, randomly oriented and beadless morphology mimicking the ECM, with an average diameter of approximately 234.172 ± 98.234 nm. The porosity of the nanofibers was in the range of 60–90%, suggesting that these nanofibers are suitable for sufficient exchange of gases and nutrients during the skin wound healing process. The FTIR and XRD data confirmed the successful formulation of gelatin-based nanofibers loaded with quercetin and ciprofloxacin [79].

The in vitro drug release studies at physiological conditions (pH 7.4 and 37 °C) showed that the release of the loaded drugs from gelatin-based hybrid nanofibers was biphasic in nature, with an initial rapid drug release followed by a slow and sustained release. The antioxidant experiments of the dual drug-loaded nanofibers using DPPH reduction methods showed significantly high antioxidant effects with more than 100% cell viability of 3T6-Swiss albino fibroblasts, indicating good biocompatibility. The in vitro antibacterial analysis showed a wide zone of inhibition against *S. aureus* resulting from the initial rapid release of ciprofloxacin. The plain nanofiber did not exhibit any significant antibacterial activity. The in vivo wound healing studies using Wistar rats showed that the full-thickness wounds treated with dual drug-loaded nanofibers provided 100% wound closure on day 16, whereas the wound closure for the plain PCL-GE and gauze was 78.85 and 71.32%, respectively [79].

Bakhsheshi-Rad et al., developed electrospun hybrid nanofibers that are based on gelatin cephalexin and PCL. The nanofibers were loaded with cephalexin for antimicrobial wound management [80]. The SEM micrographs of the nanofibers displayed a continuous, uniform, and smooth morphology with mean fiber diameters that ranged between 280 and 330 nm. The mechanical characterization of the nanofibers demonstrated a tensile strength of 3.05 MPa and a tensile strain of 89% simulating human skin. The water contact angle studies showed the hydrophilic nature of hybrid nanofibers with a contact angle of about 57 ± 1°. The in vitro antibacterial studies of cephalexin-loaded hybrid nanofibers showed a high size of inhibition of 6.1 mm for *E. coli* and 6.9 mm for *S. aureus*. The in vivo wound closure experiments employing BALB/c mice showed enhanced wound healing with the cephalexin-loaded hybrid nanofibers [80]. Adeli-Sardou et al., synthesized gelatin-PCL nanofibers loaded with Lawsone for skin regeneration. The mechanical studies of the nanofibers showed the highest tensile strength, Young’s modulus, and strain 2.14 ± 0.3 MPa, 2.12 ± 0.9 MPa, and 37 ± 6.6%, respectively. The in vitro drug release studies at physiological conditions showed prolonged release of lawsone from the hybrid nanofiber for 20 days. The in vivo wound healing studies showed that the wounds treated with lawsone-loaded nanofibers were 100% closed at the end of 14 days, while those dressed with the plain nanofibers induced 71% wound closure [81].

İnal and Mülazımoğlu reported electrospun nanofibers prepared from gelatin and poly([2-(methacryloyloxy)ethyl] trimethylammonium chloride) (PMETAC) for wound care. The degradation experiments using an environment simulating the skin demonstrated fast biodegradation of over 80% of the gelatin-PMETAC nanofibers in the first week. The in vitro cytotoxicity studies using MTT assay showed low toxicity of nanofibers when incubated with L929 fibroblast cells. The in vitro antibacterial studies of nanofibers showed high inhibition of more than 90% against *E. coli*, *S. aureus*, and *Acinetobacter baumannii*, while the inhibition for *methicillin-resistant Staphylococcus aureus* (MRSA) was 75%. These excellent antibacterial effects were induced by the presence of PMETAC [82]. Cam et al., formulated gelatin-bacterial cellulose nanofibers loaded with glibenclamide and metformin for diabetic wound care. The cytotoxicity studies showed a high cell viability of L929 mouse fibroblast cells when immersed with the nanofibers, suggesting non-toxicity and good biocompatibility. The wound contraction experiments in vivo using wounds in type-1 diabetic rats showed that the drug-loaded nanofibers exhibited superior wound healing than the pristine nanofibers on day 14, suggesting that the loading of glibenclamide and metformin into the wound dressing significantly accelerated diabetic wound healing [83].

Rather et al., prepared electrospun gelatin-PCL nanofibers functionalized with cerium oxide (CeO_2_) nanoparticles for wound healing applications. The FTIR and XRD data confirmed the successful formulation of nanofibers. The cell proliferation assay of the hybrid nanofibers exhibited high proliferation and viability of 3T3-L1 cells, indicating their good biocompatibility. The gelatin-PCL nanofibers functionalized with cerium oxide nanoparticles showed better scavenging potential when compared to the pristine nanofibers, confirming excellent antioxidant efficacy useful in the inflammatory phase of wound healing [84]. Alishahi et al., designed glucantime-loaded electrospun hybrid nanofibers that are based on gelatin and PVA/PEO/chitosan for wound care of cutaneous *Leishmania* wounds. The results from this study showed that 4 and 6 cm^2^ of drug-loaded hybrid nanofibers destroyed leishmania promastigotes up to 78% with high cell viability of fibroblast cells, indicating that the scaffolds are promising scaffolds for the management of Leishmania wounds [85].

Zhang et al., prepared gelatin-silk fibroin electrospun nanofibers encapsulated with astragaloside IV for wound management [86]. The porosity of the plain hybrid nanofibers and drug-loaded hybrid nanofibers was 88% and 89%, respectively, which are appropriate for an ideal wound dressing. The drug release profile of astragaloside IV-loaded nanofibers was rapid in the first 12 h followed by a slow-release, which is suitable for wound healing. The wound healing studies showed that the drug-encapsulated nanofiber dressings promoted a significantly higher rate of healing at the early stage of trauma than the blank nanofiber dressing and pure astragaloside IV solution group. The above-mentioned findings result from the good biocompatibility and efficient barrier against microorganisms by the astragaloside-loaded hybrid nanofibers [86]. Xia et al., designed gelatin-PCL nanofibers loaded with ciprofloxacin using centrifugal spinning for antimicrobial wound management. The water contact angle study showed the average contact angles of hybrid nanofibers were 75°, 72°, 68°, and 47° when ciprofloxacin concentration was 6%, 8%, 10%, and 12%, respectively, showing that the nanofibers become significantly more hydrophilic as the concentration of the antibiotic increased. The in vitro drug release experiments under physiological conditions showed a sustained release of ciprofloxacin from the hybrid nanofibers with a high zone of inhibition in vitro against *S. aureus* and *E. coli*. The pristine nanofiber did not display any significant antimicrobial activity [87].

Fallah et al., formulated electrospun gelatin-PCL hybrid nanofibers loaded with curcumin for antibacterial wound healing. The antimicrobial studies showed that the nanofibers displayed almost 100% antibacterial activity against MRSA and 82.56% against extended-spectrum β lactamases (ESBL), dangerous nosocomial bacterial strains. These results suggest that curcumin-loaded hybrid nanofibers are potential materials for the management of bacteria-infected wounds [88]. Gharaie et al., formulated gelatin-chitosan-PU nanofibers via the electrospinning method. The FTIR data showed the expected functional groups of the hybrid nanofibers confirming the successful formulation of the nanofiber. The SEM micrographs of the hybrid nanofibers displayed uniform and beadless morphology that mimic the ECM [89]. Pavliňáková et al., prepared hybrid nanofibers from gelatin and PCL and reinforced them with halloysite nanotubes HNTs for wound healing application. The mechanical characterizations showed that the incorporation of HNTs significantly improved the mechanical properties of hybrid nanofibers. The in vitro cytocompatibility studies showed high cell viability and proliferation of NIH-3T3 fibroblasts when incubated with HNT-reinforced nanofibers, suggesting that these scaffolds are biocompatible [90].

Jiang et al., prepared electrospun gelatin-PCL nanofibers loaded with palmatine for wound management. The in vitro experiments of the palmatine-loaded nanofibers exhibited good antioxidant and antibacterial activity. The in vivo examinations and histological studies showed that palmatine-loaded nanofibers accelerated the healing process of the full-thickness wounds and hindered hypertrophic scar development in the rabbit ear model [91].

Yang et al., reported gelatin-PCL nanofibers incorporated with Au nanoparticles for bacteria-infected wound care. The in vitro antimicrobial analysis of the nanoparticle-loaded hybrid nanofibers demonstrated excellent antibacterial efficacy against multi-drug resistant (MDR) *S. aureus* and *E. coli*. The in vivo wound healing experiments using full-thickness wounds treated with MDR *E. coli* and MDR *P. aeruginosa* on Wistar rats showed that the levels of bacteria in the bacteria-infected injuries treated with Au nanoparticle-loaded nanofibers significantly decreased with superior wound healing mechanism when compared to the pristine nanofibers and gauze [92]. Ong et al., reported Tegaderm-gelatin-PCL nanofibers for the management of wounds. The in vivo wound healing experiments using full-thickness wound on pig model demonstrated that the wounds treated with Tegaderm-gelatin-PCL nanofibers significantly accelerated reepithelization with complete lesion closure on day 28 [93]. Hivechi et al., formulated biaxially electrospun gelatin-PCL nanofibers reinforced with cellulose nanocrystals for wound healing applications [94]. The FTIR and XRD spectrums confirmed the successful formulation of nanocrystal-loaded hybrid nanofibers by displaying expected functional groups and amorphous nature, respectively. The mechanical studies showed that the incorporation of cellulose nanocrystals significantly increased the tensile strength from 14.5 ± 1.9 MPa to 23.6 ± 5.6 MPa and modulus from 112.4 ± 32.0 MPa to 206.0 ± 27.7 MPa. The wound healing analysis in vivo displayed 98 ± 3% wound closure on the 14th day for cellulose nanocrystal-loaded nanofibers while the control induced only 82 ± 6% wound closure [94].

Jafari et al., formulated bilayer gelatin-PCL nanofibers co-loaded with amoxicillin and ZnO nanoparticles for full-thickness wound management. The in vitro drug release profile showed a sustained release of amoxicillin for six days. The cytocompatibility studies in vitro using the MTT assay displayed a high cell proliferation and viability of Wharton’s jelly derived mesenchymal stromal cells (WJ-MSCs) when seeded with the nanofibers confirming good biocompatibility of the bilayered scaffolds. The in vivo wound healing studies showed a high wound closure percentage for the wounds treated with dual drug-loaded nanofibers when compared to the control [95]. Bazmandeh et al., developed formulated electrospun gelatin-chitosan-HA hybrid nanofibers for wound care. The in vitro cytotoxicity studies of the nanofibers using MTT assay demonstrated 96% cell viability of the normal human dermal fibroblasts (NHDFs) with high cell proliferation and adhesion, revealing excellent biocompatibility and non-toxicity of the nanofibers. The in vivo wound healing studies employing Wistar rats with surgical operated full-thickness incision wounds demonstrated that the wound closure in gelatin-chitosan-HA nanofibers was significantly high when compared to the gelatin-chitosan scaffolds. On day 14 post-treatment, the wounds were almost completely closed in the animal group treated with gelatin-chitosan-HA nanofibers [96].

Yao et al., developed gelatin-collagen bilayer nanofibers enriched with *Lithospermi radix* plant extract for the treatment of wounds. The in vitro drug release profile showed that the *Lithospermi radix* extract was effectively released slowly from the hybrid nanofibers. The examination of the wound-healing efficacy of the bilayer nanofibers showed the highest wound recovery rate in vivo in SD rats [97]. Hadisi et al., formulated gelatin-oxidized starch loaded with *Lawsonia Inermis* (henna) for the treatment of burn wounds [98]. The SEM images of the hybrid nanofibers displayed continuous, uniform, bead-free fibers with an average fiber diameter of about 87.01 ± 2.044 nm. The in vitro antimicrobial studies exhibited a high inhibition zone around the henna-loaded hybrid nanofibers against *S. aureus* and *E. coli*. In contrast, the plain hybrid nanofibers did not exhibit significant antibacterial effects. The in vivo wound healing studies revealed accelerated wound closure for henna-loaded nanofibers with the absence of detrimental suppurative reaction at the burn wound site. The gauze and plain nanofiber did not accelerate the wound reduction [98]. Ahlawat et al., formulated gelatin-PVA nanofibers loaded with *Carica papaya* plant extracts for microbial wound healing applications [99]. The in vitro cytotoxicity experiments showed an 80% cell viability of NIH 3T3 fibroblast cells when immersed with *Carica papaya*-loaded nanofibers revealing that hybrid nanofiber did not induce toxicity. The antimicrobial studies using the agar disc diffusion method demonstrated that the *Carica papaya*-enriched nanofibers possessed a higher zone of inhibition on agar plate against *E. coli* and *S. aureus*, suggesting good antibacterial effects of the plant extract [99].

Sobhanian et al., formulated collagen-grafted PVA-gelatin-alginate nanofibers via the electrospinning method for wound treatment. The swelling percentage of collagen-grafted hybrid nanofiber was 624.08 ± 110% and 801.25 ± 41% for 1 and 24 h, respectively, which is appropriate for cell attachment. The mechanical analysis of nanofibers displayed an ultimate tensile strength of 4.3 MPa and Young’s modulus of 150 MPa, which are in the range of the human skin. The in vitro cell proliferation evaluation of the collagen-grafted PVA-gelatin-alginate nanofibers showed high proliferation and adhesion of the fibroblasts [100]. Vázquez et al., reported gelatin-PLGA nanofiber wound dressings. The in vitro studies showed non-toxicity and high proliferation of mesenchymal stem cells (MSCs) [101]. The Ag sulfadiazine-loaded gelatin-PU nanofibers formulated by Heo et al., exhibited accelerated wound healing of burn injury in Sprague Dawley (SD) rats with good antibacterial efficacy than the gauze [101].

Other various nanofibrous scaffolds display the same properties as nanofibers. Those scaffolds include nanofibrous mats, nanofibrous membranes, nanofiber patches, nanofibrous films, nanofibrous bandages, etc. They also mimic ECM, making them suitable for wound healing and tissue regeneration applications [102]. Farzamfar et al., developed electrospinning gelatin-PCL nanofibrous mats encapsulated with taurine for wound dressing [103]. The SEM micrograph images of taurine-loaded nanofiber mats showed non-woven porous morphology with bead-free fibers. The mechanical analysis of the nanofiber mats showed ultimate tensile strengths that ranged between 2.36 ± 0.10 MPa and 2.58 ± 0.40 MPa, revealing that these scaffolds can withstand the forces exerted to the dressings during their wound healing applications. The in vitro cell proliferation studies using the MTT assay showed the high proliferation of L929 fibroblast cells when incubated with nanofiber mats, indicating good biocompatibility. The in vivo wound healing experiments using Wistar rats showed that full-thickness excisional wounds dressed with the taurine-loaded nanofibrous mats achieved a significant injury closure of 92% after 14 days when compared to the gauze that achieved 68% of wound closure [103].

Unalan et al., reported electrospun gelatin-PCL nanofibrous mats loaded with clove essential oil for antibacterial wound care management. The successful incorporation of essential oil was confirmed by FTIR analysis and gas chromatography-mass spectrometry (GC-MS). The in vitro cytotoxicity studies showed high cell viability and growth of NHDFs when incubated with the nanofiber mats for two days, suggesting non-toxicity and excellent biocompatibility of the mats. The antimicrobial studies of nanofibrous mats loaded with clove essential oil showed high inhibition effects against *E. coli* and *S.aureus* than the pristine hybrid nanofiber mats, revealing nanofibrous as potential antibacterial wound dressing material. Furthermore, the in vitro wound healing studies of the essential oil-loaded nanofiber mats using scratch assay showed an accelerated wound closure mechanism [104]. Ramalingam et al., fabricated electrospun nanofibrous gelatin-PCL mats enriched with *Gymnema sylvestre* plant extract [105]. The in vitro drug release profile showed an initial burst release of plant extract that can result in preventing the colonization of bacteria. The in vitro experiments showed that the electrospun hybrid mats stimulated cellular adhesion, migration, and proliferation of human dermal fibroblasts and keratinocytes, which are vital cell types involved in the skin healing process. The in vitro antimicrobial studies using the Kirby-Bauer disc diffusion assay showed no zone of inhibition around the plain nanofibrous mats, whereas the mats loaded with *Gymnema sylvestre* extracts showed a clear zone of inhibition against *E. coli*, *S. aureus*, MRSA, and *P. aeruginosa* [105].

Salehi et al., reported nanofibrous mats prepared from gelatin, PCL and enriched with cinnamon for wound treatment. The in vivo wound healing study using a full-thickness model on Wistar rats showed an accelerated wound healing process when injuries were dressed with cinnamon-loaded mats compared to the plain mats [106]. The electrospun cellulose acetate-gelatin-hydroxyapatite nanofiber mats reported by Samadian et al., demonstrated that all the fabricated mats possessed a higher wound closure percentage on the full-thickness excision wound model in male Wistar rats than the sterile gauze [107].

Zahiri et al., prepared nanofiber gelatin-PCL mats incorporated with curcumin-loaded chitosan nanoparticles for application in wound treatment. The in vitro drug release studies at physiological conditions showed sustained and controlled release of nanocurcumin from the hybrid nanofibers. The in vivo wound healing studies of curcumin-loaded hybrid nanofibers showed high degrees of wound healing with more than 62% and 82% wound closure on days 7 and 14, respectively [108]. Yao et al., formulated gelatin-keratin nanofibrous mats for wound dressing. The SEM images of the hybrid mats exhibited a uniform morphology and bead-free structure with a mean fiber diameter of 160.4 nm. The in vitro cell proliferation studies showed high proliferation and adhesion of L929 fibroblasts when incubated with the nanofiber mats, indicating non-toxicity. The in vivo experiments using full-thickness wounds in rats showed a 97.9 ± 1.6% closure in wound area on day 14 post-surgery, whereas the gauze displayed only 85.8 ± 6.0% wound closure [109]. Basar et al., formulated gelatin-PCL nanofiber mats for the controlled release of ketoprofen. The in vitro drug release data demonstrated a burst release of ketoprofen that reached a plateau from the nanofibrous mats followed by a slow and sustained release [110].

Amer et al., fabricated gelatin-PVA nanofibrous mats incorporated with Ag nanoparticles for wound healing application. The FTIR data confirmed the successful preparation of Ag nanoparticle-loaded mats by showing the expected functional groups. The SEM images of the hybrid nanofiber mats showed a combination of smooth, beads-free, and uniform fibrous morphologies. The in vivo studies showed an accelerated wound healing process when the full-thickness skin wounds in rabbits were treated with both hybrid nanofiber mats incorporated with nanoparticles and pristine hybrid nanofiber mats [111]. Cai et al., prepared nanofibrous membranes that are based on gelatin and PCL. The membranes were loaded with Fe_3_O_4_ nanoparticles for microbial-infected wound care [112]. The SEM morphology of hybrid fiber membranes was smooth without noticeable beads with a diameter of 435 nm, suggesting that all the Fe_3_O_4_ nanoparticles were successfully loaded in the nanofibrous membranes. The mechanical analysis of 1 wt% nanoparticle-loaded nanofibrous scaffolds showed a tensile strength of 6.4 ± 0.2 MPa, Young’s modulus of 124.9 ± 4.8 MPa, Elongation at break of 8.2 ± 0.3%, and toughness of 0.26 ± 0.03 MJ/m^3^. The in vitro antimicrobial experiments of nanoparticle-incorporated nanofibers demonstrated a high zone of inhibition against *S aureus* and *E. coli*, indicating that these scaffolds could be effective antibacterial wound dressings [112].

Shi et al., developed gelatin-PCL nanofiber membranes encapsulated with Trimethoxysilylpropyl octadecyldimethyl ammonium chloride (QAS) for antibacterial wound management [113]. The physicochemical properties of QAS-loaded hybrid nanofibrous membranes were confirmed by FTIR and X-ray photoelectron spectroscopy (XPS). The water uptake studies showed that all the formulated membranes have a mean water absorption of above 400% that meets the condition of an ideal wound dressing. The mechanical analysis showed that the nanofiber membrane tensile strength ranged between 9 and 12 MPa [113]. The in vitro antibacterial studies of QAS-loaded hybrid nanofibrous membranes showed a 99.9% reduction of both *S. aureus* and *E. coli* after 6 h with a high cell viability of more than 90% when incubated with L929 fibroblast cells. These results revealed that QAS-loaded nanofibrous membranes are potential materials for the treatment of bacteria-infected wounds with excellent biocompatibility and non-toxicity [113].

Semnani et al., formulated gelatin-PVP nanofibrous membranes loaded with Ag sulfadiazine for bacteria-infected wound treatment. The mechanical analysis of Ag sulfadiazine-loaded nanofiber membranes showed Young’s modulus of 22.58 MPa that was in the same range as human skin. The in vitro antimicrobial experiments showed high inhibition zone against *E. coli* and *S. aureus* when incubated with Ag sulfadiazine-loaded nanofiber membranes while pristine membranes did not demonstrate any antibacterial effects as expected. Moreover, the in vitro drug release profile under physiological conditions showed that Ag sulfadiazine release behavior from the fabricated membranes was short-term [114]. Eskandarinia et al., fabricated nanofibrous gelatin-PCL-PU membrane scaffolds loaded with propolis for wound management. The in vitro antibacterial studies of propolis-loaded nanofibrous membranes showed superior antibacterial effects against *E. coli* (1.9 ± 0.4mm), *S. epidermidis* (1.0 ± 0.2 mm), and *S. aureus* (5.4 ± 0.3 mm). The wound healing analysis demonstrated that the nanofibrous membranes significantly accelerated the wound reduction and collagen deposition in the Wistar rats’ skin wound model [115].

Ogawa et al., formulated nanofibrous films that are based on gelatin and chitin for wound management. The nanofiber films displayed transmittances that are greater than 75% at 600 nm, indicating good transparency that is appropriate for wound monitoring without removing the nanofibrous film. The biocompatibility studies in vivo showed many neutrophils in the subcutaneous layer of mice when treated with the films, suggesting non-toxicity [116]. Naseri-Nosar et al., formulated gelatin-PCL hybrid nanofibrous films incorporated with CeO_2_ nanoparticles for wound treatment. The in vivo studies using the rat model showed that the full-thickness wounds dressed with the CeO_2_ nanoparticle-loaded film dressings achieved a more significant wound closure of almost 100% after two weeks than the sterile gauze with only 63% wound closure [117].

Josh et al., developed heparin-functionalized nanofibrous patches from gelatin and PCL co-loaded with bFGF and VEGF for skin regeneration [118]. The SEM micrograph images of nanofiber patches showed a beadless uniform nanofiber structure with an average fiber diameter of approximately 370 nm. The water vapor transmission studies of gelatin-PCL nanofiber patches exhibited a WVTR value of about 2467 ± 243 g/m^2^/day, indicating that the formulated materials are ideal wound dressings. The in vivo wound healing experiments using the rat model demonstrated that the heparin-functionalized dual GF-loaded nanofiber patches significantly accelerated the wound healing process with complete regeneration of the skin and the absence of scarring at the end of 14 days. The pristine patches wounds were not completely healed [118]. Lv et al., prepared gelatin-PCL nanofibrous composite materials loaded with silicate-based bioceramic particles for diabetic wound management [119]. The in vivo wound healing experiments using the diabetic mice model demonstrated that the diabetic wounds treated with bioceramic particle-loaded nanofibrous composite healed significantly faster after seven days than the plain nanofibrous composites and control.

The electrospinning technique was used to fabricate gelatin-based hybrid nanofibrous scaffolds due to its simplicity and cost-effectiveness when compared to other known methods. The SEM micrographs of gelatin nanofibers exhibited a randomly oriented and beadless morphology mimicking the ECM, indicating that these nanofibers can provide an appropriate environment for the wound healing process. Loading gelatin nanofibers with bioactive agents also promote their antibacterial efficacy, making them ideal wound dressings. The drug-loaded gelatin-based hybrid nanofibers demonstrated sustained drug release profiles with excellent biological effects (e.g., antibacterial activity) and accelerated the wound healing process of chronic injuries. The biological outcomes of gelatin-based nanofibers demonstrate that they are suitable for the management of chronic wounds, such as diabetic wounds, full-thickness wounds, etc.

### 5.5. Gelatin-Based Microspheres

**Figure 9:**
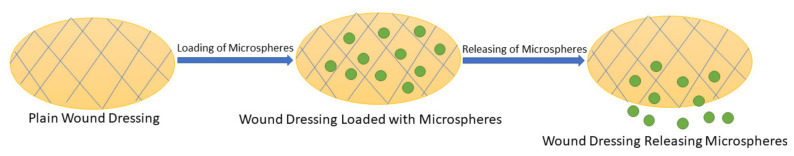
Schematic Diagram of Wound Dressing loaded with Microspheres.

Microspheres are spherical shells that are commonly formulated from biodegradable or resorbable polymers. They possess a very small diameter, generally in the micrometre range [120]. This type of system displays unique features, such as high drug load capacity, site-specific action, controlled drug release, and good stability (thermally, physically, and chemically). They are also non-toxic, cost-effective, and easy to prepare [121,122]. Gelatin-based microspheres have been reported to display distinct properties appropriate for wound dressings (Table 5). The advantages of gelatin-based wound dressings are summarized in Table 5. These microspheres can be further loaded in various types of wound dressing scaffolds (Figure 9). Che et al., incorporated gelatin microspheres into a composite hydrogel for improved mechanical properties. The gelatin microspheres were developed by an emulsion cross-linking method before incorporation into the hydrogel. Increasing the ratios of microspheres to 40 mg/mL resulted in a short gelation time and low swelling capability of the hydrogel with high mechanical strength. However, the hydrogel incorporated with 30 mg/mL of the gelatin microspheres displayed good stability and mechanical properties appropriate for wound healing with potent bacteria growth inhibition effects against *Escherichia coli* and *S. aureus* [123]. 

Koslowska et al., prepared microspheres from gelatin and a combination of collagen and gelatin. The microspheres were loaded into collagen/gelatin/hydroxyethyl cellulose composites. The composites were loaded with *calendula officinalis* flower extract. The incorporation of the microspheres into the composites enhanced the porosity, drug loading capability, and drug release of the composites [124]. Fang et al., incorporated gelatin microspheres loaded with ciprofloxacin hydrochloride into chitosan/gelatin composites. The wound dressings displayed appropriate porosity, excellent mechanical properties, high water absorption ability, and biocompatibility. The drug release profile was sustained with a good antibacterial effect against *E. coli*, *P. aeruginosa,* and *S. aureus*, in vitro and in vivo. The deposition of collagen was accelerated revealing microspheres as potential wound dressing for the treatment of bacterial-infected wounds and wounds with seawater immersion [125]. Thyagarajan et al., prepared wound dressings that will inhibit overexpression of matrix metalloproteinase, a family of endopeptidases involved in the remodelling of ECM, which is also capable of degrading ECM when over-expressed. Microspheres were prepared from gelatin and siderophore. Siderophores are iron chelators that inhibit matrix metalloproteinase at the wound site and also reduce bacterial load. The microspheres were rigid with high porosity, and a mean diameter in the range of 7.0 ± 0.52–25.3 ± 0.31 µm. The microspheres were non-toxic, biocompatible on NIH 3T3 fibroblast cell lines with a rapid drug release profile, and supported cell attachment and proliferation [126]. Yang et al., loaded platelet-rich plasma into gelatin microspheres prepared by an emulsion cross-linking method. The average particle size of the microspheres was 15.95 ± 3.79 μm. The drug release profile of the microsphere was sustained for seven days. The microspheres promoted cell proliferation and migration of L929 mouse fibroblast cells in vitro [127].

Li et al., reported gelatin-based injectable microspheres for bleeding wounds. The microspheres were prepared by a water-in-oil emulsion method, followed by crosslinking with glutaraldehyde. The microspheres were negatively charged and the blood clotting time of the microspheres occurred within 60 s. In vivo hemostatic studies on a deep liver wound bleeding model showed a good hemostatic effect of the microspheres. The formulations are suitable for deep wound bleeding in surgery [128]. Zhu et al., prepared collagen/cellulose nanocrystals incorporated with gentamycin sulfate loaded in gelatin microsphere. The in vitro release profile of the drug was sustained for 144 h. The scaffold displayed good compatibility with NIH-3T3 cells in vitro and was effective against *E. coli* and *S. aureus* [129]. The wound dressings are potential wound dressings for the treatment of microbial-infected burn wounds. Kirubanandan et al., developed porous collagen scaffolds incorporated with ciprofloxacin-loaded gelatin microspheres. The delivery of ciprofloxacin from the scaffolds was controlled for two days with 27% drug burst release within the first 5 h. The scaffolds inhibition effect against pseudomonas pathogens was significant in vitro. In vivo study of the scaffolds in full-thickness wounds revealed accelerated healing in 20 days. The wound closure was confirmed by epidermis and dermis regeneration at the wound site. The ciprofloxacin-loaded gelatin microspheres in the scaffold promoted a sustained release profile of the drug via degradation of gelatin in the infected wound environment [130].

Gelatin-based microspheres were prepared and loaded with bioactive agents, such as antibiotics and growth factors before incorporation into the scaffolds. The biodegradable nature of gelatin promoted a sustained release profile, as well as increased the water absorption capacity and the hemostatic effect of the wound dressing. Based on the different research reports, gelatin-based microspheres are suitable for the treatment of burn and infected wounds, and also appropriate for skin regeneration.

## 6. Gelatin-Based Hybrid Wound Dressings vs Traditional Wound Dressing Technology

The currently used traditional dressings include plasters, gauze, cotton wool, tulle, bandages, and lint, which are utilized as primary or secondary dressings to protect injuries from contaminations [131]. The other advantages of traditional wound dressings include their ability to absorb wound exudate, offer a dry environment for the wound, and cushion the wound [131,132]. Gauze products that are formulated from non-woven and woven fibers of rayon, polyesters, and cotton can provide a limited barrier against bacterial invasion. Cotton bandages are usually employed for the retention of light wound dressings, short-stretch compression, and high compression bandages offer continuous compression in venous ulcers. Tulle wound dressings (e.g., Paratulle, and Jelonet) are commercially available and are appropriate for superficial clean injury [3]. Although traditional wound dressing products exhibit these interesting advantages, they suffer from several limitations. The disadvantages of traditional dressings include their inability to provide moisture to the wound bed for accelerated wound healing and the capability to cause further skin damage or pain during removal resulting from their high adherent when used in high exuding wounds [133]. They display poor vapor transmission, cause bleeding, and harm the newly formed epithelium during removal. The leakage of wound exudates from traditional wound dressings promotes bacterial invasion [134].

The gelatin-based hybrid wound dressings can be used as ideal dressings to replace the traditional wound dressing products because of their interesting features when compared to the traditional dressings. Gelatin hybrid dressings provide a moist environment for injuries to recover quickly. The suitably moist environment that is offered by gelatin hybrid dressings is due to their moderate WVTR. Interestingly, gelatin-based wound dressings can be loaded with various types of bioactive agents (e.g., antibiotics, nanoparticles, microspheres, antioxidants, etc.) to improve their biological activities and speed up the wound healing process that is essential in the treatment of chronic wounds. Gelatin-based hybrid wound dressings also display good mechanical properties, excellent biocompatibility, non-toxicity, good biodegradation, high porosity, and good absorption and swelling capacity. Nevertheless, gelatin dressings suffer from poor antibacterial activity that is overcome by encapsulating selected antimicrobial agents (such as ciprofloxacin, essential oils, and metal-based nanoparticles) into them. Gelatin-based hybrid wound dressings demonstrate many distinct advantages when compared to the traditional dressings, making them promising scaffolds for the treatment of chronic and high exuding wounds.

## 7. Conclusions and Future Perspectives

Gelatin-based hybrid wound dressings promote accelerated wound healing and also exhibit ideal properties, such as moderate WVTR, high porosity, good mechanical performance, high water uptake and swelling behavior, good biocompatibility, antigenicity, and non-toxicity. However, the application of gelatin alone for the development of wound dressings is hampered by its poor antibacterial activity and weak mechanical performance. It is used in combination with other polymers, resulting in excellent mechanical properties that are required for ideal wound dressings. To further improve the therapeutic outcomes of gelatin-based hybrid scaffolds, they are loaded with bioactive agents. The in vitro and in vivo studies have demonstrated that the poor antimicrobial properties of gelatin are overcome by combining this biopolymer with other polymers and loading them with antibacterial agents. The loading of two bioactive agents into gelatin-based hybrid scaffolds resulted in superior biological efficacy due to synergistic effects. Interestingly, the hydrophilic nature of gelatin-based hybrid dressings does not only promote cell adhesion but also stimulates cell differentiation and proliferation, which offers a good environment for wound healing and skin regeneration. There are very few gelatin-based hybrid dressings that are under clinical trials, but many reports of in vitro and in vivo studies have shown that these hybrid dressings are promising wound dressings. Most of the reported gelatin-based wound dressings are still in the preclinical phase.

Different strategies, such as incorporating bioactive agents (such as nanoparticles, plant extracts, antibiotics, growth factors, etc.) into the prepared wound dressings, enhanced their bactericidal activity and wound healing effects. Despite the level of development of gelatin-based wound dressings attained so far, there is a pressing need for further improvements of these dressings, such as the incorporation of antibacterial agents, nanoparticles with antibacterial agents, growth factors with antibacterial agents, etc., to afford improved therapeutic outcomes. The use of gelatin biopolymers in combination with selected synthetic polymers for 3D printing to produce new wound dressing products is an alternative approach that should be explored for the preparation of effective wound dressings. The incorporation of sensors into these wound dressings for monitoring infected wounds and the healing phases is also a potential approach to developing effective wound dressings. The knowledge acquired by researchers so far on wound healing and the trend of research, together with the use of nanotechnology, represent tools that will result in effective wound dressings for treating chronic wounds.

## Figures and Tables

**Table 1:** Summary of Gelatin-based Hydrogel Wound Dressings.

Types of Wound Dressings	Polymers Combined with Gelatin	Loaded Bioactive Agents	Outcomes	References
Hydrogels	HA	Recombinant thrombomodulin	High swelling capacity and accelerated diabetic wound closure.	[38]
Hydrogels	Oxidized Starch	_	Good cytocompatibility and fast wound healing mechanism with less scar development.	[39]
Hydrogels	Pluronic	Nanocurcumin	Accelerated burn wound reduction.	[40]
Hydrogels	Gellan	Tannic acid	Superior antimicrobial activity and fast full-thickness wound healing.	[41]
Hydrogels	PEG	ASCs	Non-toxicity on skin cells and fast wound contraction.	[42]
Hydrogels	Bacterial cellulose	Curcumin	Good mechanical properties and controlled drug release.	[43]
Hydrogels	Bacterial cellulose	Methylene blue	Good mechanical performance and high swelling capacity.	[44]
Hydrogels	PEG and CMC	_	Excellent swelling behavior	[45]
Hydrogels	PVA and chitosan	_	Excellent mechanical properties and good hemostatic effects.	[46]

**Table 2:** Summary of Gelatin-based Hybrid Film and Membrane Wound Dressings.

Types of Wound Dressings	Polymers Combined with Gelatin	Loaded Bioactive Agents	Outcomes	References
Films	Chitosan	Tannic acid and bacterial nanocellulose	Good mechanical performance and faster full-thickness wound closure.	[49]
Films	Chitosan	Allantoin	Excellent biocompatibility and non-toxicity with superior antibacterial efficacy.	[50]
Films	Chitosan	Nanocrystalline cellulose and calcium peroxide	Moderate WVTR and excellent cytocompatibility	[51]
Films	Chitosan	Lupeol	Initial rapid drug release followed by sustained release, and good antioxidant efficacy.	[52]
Films	Alginate	Hydroxyapatite	High antibacterial activity.	[53]
Films	Chitosan	ZnO nanoparticles	Good antibacterial effects and increased full-thickness wound contraction rate.	[54]
Films	Chitosan	Bone ash and ciprofloxacin	Superior antimicrobial effects	[55]
Films	PCL	Catechin	High cell and proliferation of skin cells.	[56]
Films	Chitosan	_	High swelling capacity and accelerated wound healing.	[57]
Films	Cellulose	_	Excellent fluid absorbing effect and fast wound closure.	[58]
Membranes	CM chitosan and HA	_	High cell viability and proliferation	[60]
Membranes	Chitosan	_	High growth inhibition against several bacterial strains	[61]
Membranes	Polymyxin B sulfate	Ciprofloxacin and HNTs	High swelling capacity, non-toxic, and good antibacterial activity.	[62]

**Table 3:** Summary of Gelatin-based hybrid Sponge Wound Dressings.

Types of Wound Dressings	Polymers Combined with Gelatin	Loaded Bioactive Agents	Outcomes	References
Sponges	Konjac glucomannan	Au nanoparticles and gentamicin	Non-toxic and superior antibacterial activity with accelerated full-thickness wound healing.	[66]
Sponges	Collagen	bFGF	The faster full-thickness wound healing process.	[67]
Sponges	Gelatin	Ag nanoparticles	High porosity and high antibacterial efficacy.	[68]
Sponges	Chitosan	Curcumin	Initial burst drug release followed by sustained slowly release with fast wound healing and no scar formation	[69]
Sponges	Chitosan	Tannis and PRP	Accelerated wound healing	[70]
Sponges	Keratin and fibrin	Mupirocin	Initial burst drug release followed by sustained slow release with good antibacterial efficacy.	[71]
Sponges	Bacterial cellulose	Ampicillin	Sustained drug release and excellent antibacterial activity.	[72]
Sponges	Sodium alginate	Tetracycline hydrochloride	Good swelling behavior and controlled drug release with high antibacterial effects.	[73]

**Table 4:** Gelatin-based hybrid nanofiber Wound Dressings.

Types of Wound Dressings	Polymers Combined with Gelatin	Loaded Bioactive Agents	Outcomes	References
Nanofibers	PCL	Ciprofloxacin	Good porosity, sustained drug release, and accelerated full-thickness wound closure.	[79]
Nanofibers	PCL	Cephalexin	Good mechanical properties and high antibacterial activity.	[80]
Nanofibers	PCL	Lawsone	Prolonged drug release and accelerated wound reduction	[81]
Nanofibers	PMETAC	_	Non-toxicity and excellent antibacterial activity	[82]
Nanofibers	Bacterial cellulose	Glybenclamide and metformin	Good biocompatibility and quickly diabetic wound healing.	[83]
Nanofibers	PCL	CeO_2_ nanoparticles	High cell viability and proliferation with good antioxidant efficacy.	[84]
Nanofibers	PVA/PEO/chitosan	Glucantime	Destroyed leishmania promatigotes with high cell viability of skin cells	[85]
Nanofibers	Silk fibroin	Astragaside IV	High porosity and high wound closure rate	[86]
Nanofibers	PCL	Ciprofloxacin	Good antibacterial efficacy	[87]
Nanofiber	PCL	Curcumin	Excellent antibacterial activity	[88]
Nanofiber	Chitosan and PU	_	Bead-free morphology biomimicking ECM.	[89]
Nanofibers	PCL	HNTs	Enhanced mechanical properties and good cytocompatibility.	[90]
Nanofibers	PCL	Palmatine	Good antioxidant and antibacterial effects with accelerated wound healing.	[91]
Nanofibers	PCL	Au nanoparticles	Excellent antibacterial activity	[92]
Nanofibers	PCL	_	Accelerated wound healing process	[93]
Nanofibers	PCL	Cellulose nanocrystals	Excellent mechanical properties and fast wound closure rate.	[94]
Nanofibers	PCL	Amoxicillin and ZnO nanoparticles	Sustained drug release and high cell proliferation.	[95]
Nanofibers	Chitosan and HA	_	Accelerated full-thickness wound contraction.	[96]
Nanofibers	Collagen	Lithospermi radix plant extract	Higher wound recovery rate	[97]
Nanofibers	Oxidized starch	Lawsonia Inermis	Good antibacterial activity and accelerated burn wound healing.	[98]
Nanofibers	PVA	Carica papaya	Non-toxicity and good antibacterial efficacy.	[99]
Nanofibers	Collagen, alginate, and PVA	_	Good mechanical performance and high cell proliferation and adhesion	[100]
Nanofibers	PLGA	_	Non-toxicity and high cell proliferation.	[101]
Nanofibrous mats	PCL	Taurine	Good mechanical properties and accelerated wound closure.	[103]
Nanofibrous mats	PCL	Clove essential oil	High cell proliferation and good antibacterial efficacy.	[104]
Nanofibrous mats	PCL	Gymnema sylvestre plant extract	Promoted skin cellular adhesion, migration, and proliferation with good antimicrobial effects	[105]
Nanofibrous mats	PCL	Cinnamon	Accelerated full-thickness wound closure rate.	[106]
Nanofibrous mats	Cellulose	Hydroxyapatite	High wound closure percentage	[107]
Nanofibrous mats	PCL	Curcumin and chitosan nanoparticles	Sustained drug release and a high rate of wound healing.	[108]
Nanofibrous mats	Keratin	_	High cell attachment and proliferation with accelerated wound contraction.	[109]
Nanofibrous mats	PCL	Ketoprofen	Sustained drug release profile and enhance cell adhesion and proliferation.	[110]
Nanofibrous mats	PVA	Ag nanoparticles	Accelerated full-thickness wound closure	[111]
Nanofibrous membranes	PCL	Fe_3_O_4_	Good mechanical properties and potential antibacterial efficacy.	[112]
Nanofibrous membranes	PCL	QAS	Excellent biocompatibility and superior antibacterial efficacy	[113]
Nanofibrous membranes	PVP	Ag sulfadiazine	Excellent mechanical performance and superior antibacterial efficacy	[114]
Nanofibrous membranes	PCL and PU	Propolis	Accelerated wound healing	[115]
Nanofibrous films	Chitin	_	Good transparency and non-toxicity	[116]
Nanofibrous films	PCL	CeO_2_ nanoparticles	Accelerated wound closure	[117]
Nanofibrous patches	PCL	bFGF and VEGF	Fast skin regeneration skin with scar formation	[118]
Nanofibrous composite materials	PCL	Silicate-based bioceramic particles	Fast diabetic wound healing	[119]

**Table 5:** Summarizing the Advantages and Disadvantages of Gelatin-based Hybrid Wound Dressings.

Types of Gelatin-Based Hybrid Wound Dressings	Advantages	Disadvantages
Hydrogels	Ability to be loaded with bioactive agents. Accelerate wound healing process and formation of less scar. Good biocompatibility and non-toxicity. Excellent mechanical properties. Sustained and controlled drug release	The high content of gelatin can decrease hydrogel porosity. Less antibacterial activity if not loaded with bioactive agents.
Films and Membranes	Transparent Appropriate WVTR for the wound healing process. Excellent mechanical performance. Non-toxicity Sustained drug release kinetics.	Low content of gelatin can result in low absorption capacity.
Sponges	Good cytocompatibility High porosity Ability to be loaded with drugs. Capacity to accelerate wound healing process without the formation of scars. Good biological activities	Delayed wound healing process if they are not loaded with antibacterial drugs. They are not suitable for low exuding injuries due to the high swelling capacity.
Nanofibers	They possess a structure that mimics ECM. High porosity Ability to be used as drug delivery systems. Easily formulated by the most popular technique called electrospinning. Excellent biocompatibility	Poor antibacterial activity if not loaded with antibacterial drugs.
Microspheres	They can be loaded with bioactive agents. Display good antibacterial activity. They can be loaded onto other wound dressings to improve biological activities. They are suitable for bleeding, infected, and burn wounds.	There are no reported shortcomings

## Data Availability

The data presented in this study are available on request from the corresponding author.
